# Supplementary material for: Structure and function of the Zika virus full-length NS5 protein
Source: Nat Commun. 2017 Mar 27;8:14762. doi: 10.1038/ncomms14762 (PMC5378950; doi:10.1038/ncomms14762)
Supplement: Supplementary Information — Supplementary Figures and Supplementary Table. [file ncomms14762-s1.pdf]

Supplementary Table 1. DNA sequence synthesized to express the Zika virus NS5 protein.

MR766 NS5 sequence

```
1     ACAGAGAACAGATTGGTGGTGGAGGTGGGACGGGAGAGACTCTGGGAGAGAAGTGGAAAG
61    CTCGTCTGAATCAGATGTCGGCCCTGGAGTTCTACTCTTATAAAAAGTCAGGTATCACTG
121   AAGTGTGTAGAGAGGAGGCTCGCCGTGCCCTCAAGGATGGAGTGGCCACAGGAGGACATG
181   CCGTATCCCGGGGAAGTGCAAAGATCAGATGGTTGGAGGAGAGAGGATATCTGCAGCCCT
241   ATGGGAAGGTTGTTGACCTCGGATGTGGCAGAGGGGGCTGGAGCTATTATGCCGCCACCA
301   TCCGCAAAGTGCAGGAGGTGAGAGGATACACAAAGGGAGGTCCCGGTCATGAAGAACCCA
361   TGCTGGTGCAAAGCTATGGGTGGAACATAGTTCGTCTCAAGAGTGGAGTGGACGTCTTCC
421   ACATGGCGGCTGAGCCGTGTGACACTCTGCTGTGTGACATAGGTGAGTCATCATCTAGTC
481   CTGAAGTGGAAGAGACACGAACACTCAGAGTGCTCTCTATGGTGGGGGACTGGCTTGAAA
541   AAAGACCAGGGGCCTTCTGTATAAAGGTGCTGTGCCCATACACCAGCACTATGATGGAAA
601   CCATGGAGCGACTGCAACGTAGGCATGGGGGAGGATTAGTCAGAGTGCCATTGTGTCGCA
661   ACTCCACACATGAGATGTACTGGGTCTCTGGGGCAAAGAGCAACATCATAAAAAGTGTGT
721   CCACCACAAGTCAGCTCCTCCTGGGACGCATGGATGGCCCCAGGAGGCCAGTGAAATATG
781   AGGAGGATGTGAACCTCGGCTCGGGTACACGAGCTGTGGCAAGCTGTGCTGAGGCTCCTA
841   ACATGAAAATCATCGGCAGGCGCATTGAGAGAATCCGCAATGAACATGCAGAAACATGGT
901   TTCTTGATGAAAACCACCCATACAGGACATGGGCCTACCATGGGAGCTACGAAGCCCCCA
961   CGCAAGGATCAGCGTCTTCCCTCGTGAACGGGGTTGTTAGACTCCTGTCAAAGCCTTGGG
1021  ACGTGGTGACTGGAGTTACAGGAATAGCCATGACTGACACCACACCATACGGCCAACAAA
1081  GAGTCTTCAAAGAAAAAGTGGACACCAGGGTGCCAGATCCCCAAGAAGGCACTCGCCAGG
1141  TAATGAACATAGTCTCTTCCTGGCTGTGGAAGGAGCTGGGGAAACGCAAGCGGCCACGCG
1201  TCTGCACCAAAGAAGAGTTTATCAACAAGGTGCGCAGCAATGCAGCACTGGGAGCAATAT
1261  TTGAAGAGGAAAAAGAATGGAAGACGGCTGTGGAAGCTGTGAATGATCCAAGGTTTTGGG
1321  CCCTAGTGGATAGGGAGAGAGAACACCACCTGAGAGGAGAGTGTCACAGCTGTGTGTACA
1381  ACATGATGGGAAAAAGAGAAAAGAAGCAAGGAGAGTTCGGGAAAGCAAAAGGTAGCCGCG
1441  CCATCTGGTACATGTGGTTGGGAGCCAGATTCTTGGAGTTTGAAGCCCTTGGATTCTTGA
1501  ACGAGGACCATTGGATGGGAAGAGAAAACTCAGGAGGTGGAGTCGAAGGGTTAGGATTGC
1561  AAAGACTTGGATACATTCTAGAAGAAATGAATCGGGCACCAGGAGGAAAGATGTACGCAG
1621  ATGACACTGCTGGCTGGGACACCCGCATTAGTAAGTTTGATCTGGAGAATGAAGCTCTGA
1681  TTACCAACCAAATGGAGGAAGGGCACAGAACTCTGGCGTTGGCCGTGATTAAATACACAT
1741  ACCAAAACAAAGTGGTGAAGGTTCTCAGACCAGCTGAAGGAGGAAAAACAGTTATGGACA
1801  TCATTTCAAGACAAGACCAGAGAGGGAGTGGACAAGTTGTCACTTATGCTCTCAACACAT
1861  TCACCAACTTGGTGGTGCAGCTTATCCGGAACATGGAAGCTGAGGAAGTGTTAGAGATGC
1921  AAGACTTATGGTTGTTGAGGAAGCCAGAGAAAGTGACCAGATGGTTGCAGAGCAATGGAT
1981  GGGATAGACTCAAACGAATGGCGGTCAGTGGAGATGACTGCGTTGTGAAGCCAATCGATG
2041  ATAGGTTTGCACATGCCCTCAGGTTCTTGAATGACATGGGAAAAGTTAGGAAAGACACAC
2101  AGGAGTGGAAACCCTCGACTGGATGGAGCAATTGGGAAGAAGTCCCGTTCTGCTCCCACC
2161  ACTTCAACAAGCTGTACCTCAAGGATGGGAGATCCATTGTGGTCCCTTGCCGCCACCAAG
2221  ATGAACTGATTGGCCGAGCTCGCGTCTCACCAGGGGCAGGATGGAGCATCCGGGAGACTG
2281  CCTGTCTTGCAAAATCATATGCGCAGATGTGGCAGCTCCTTTATTTCCACAGAAGAGACC
2341  TTCGACTGATGGCTAATGCCATTTGCTCGGCTGTGCCAGTTGACTGGGTACCAACTGGGA
2401  GAACCACCTGGTCAATCCATGGAAAGGGAGAATGGATGACCACTGAGGACATGCTCATGG
2461  TGTGGAATAGAGTGTGGATTGAGGAGAACGACCATATGGAGGACAAGACTCCTGTAACAA
2521  AATGGACAGACATTCCCTATCTAGGAAAAAGGGAGGACTTATGGTGTGGATCCCTTATAG
2581  GGCACAGACCCCGCACCACTTGGGCTGAAAACATCAAAGACACAGTCAACATGGTGCGCA
2641  GGATCATAGGTGATGAAGAAAAGTACATGGACTATCTATCCACCCAAGTCCGCTACTTGG
2701  GTGAGGAAGGGTCCACACCCGGAGTGTTGTAAAAGTGGATAACGGATCCG
```

PE243/2015 NS5 sequence

```
1       ACAGAGAACAGATTGGTGGTGGGGGTGGAACAGGAGAGACCCTGGGAGAGAAATGGAAGG
61      CCCGCTTGAACCAGATGTCGGCCCTGGAGTTCTACTCCTACAAAAAGTCAGGCATCACCG
121     AGGTGTGCAGAGAAGAGGCCCGCCGCGCCCTCAAGGACGGTGTGGCAACGGGAGGCCATG
181     CTGTGTCCCGAGGAAGTGCAAAGCTGAGATGGTTGGTGGAGCGGGGATACCTGCAGCCCT
241     ATGGAAAGGTCATTGATCTTGGATGTGGCAGAGGGGGCTGGAGTTACTACGCCGCCACCA
301     TCCGCAAAGTTCAAGAAGTGAAAGGATACACAAAAGGAGGCCCTGGTCATGAAGAACCCG
361     TGTTGGTGCAAAGCTATGGGTGGAACATAGTCCGTCTTAAGAGTGGGGTGGACGTCTTTC
421     ATATGGCGGCTGAGCCGTGTGACACGTTGCTGTGTGACATAGGTGAGTCATCATCTAGTC
481     CTGAAGTGGAAGAAGCACGGACGCTCAGAGTCCTCTCCATGGTGGGGGATTGGCTTGAAA
541     AAAGACCAGGAGCCTTTTGTATAAAAGTGTTGTGCCCATACACCAGCACTATGATGGAAA
601     CCCTGGAGCGACTGCAGCGTAGGTATGGGGGAGGACTGGTCAGAGTGCCACTCTCCCGCA
661     ACTCTACACATGAGATGTACTGGGTCTCTGGAGCGAAAAGCAACACCATAAAAAGTGTGT
721     CCACCACGAGCCAGCTCCTCTTGGGGCGCATGGACGGGCCTAGGAGGCCAGTGAAATATG
781     AGGAGGATGTGAATCTCGGCTCTGGCACGCGGGCTGTGGTAAGCTGCGCTGAAGCTCCCA
841     ACATGAAGATCATTGGTAACCGCATTGAAAGGATCCGCAGTGAGCACGCGGAAACGTGGT
901     TCTTTGACGAGAACCACCCATATAGGACATGGGCTTACCATGGAAGCTATGAGGCCCCCA
961     CACAAGGGTCAGCGTCCTCTCTAATAAACGGGGTTGTCAGGCTCCTGTCAAAACCCTGGG
1021    ATGTGGTGACTGGAGTCACAGGAATAGCCATGACCGACACCACACCGTATGGTCAGCAAA
1081    GAGTTTTCAAGGAAAAAGTGGACACTAGGGTGCCAGACCCCCAAGAAGGTACTCGTCAGG
1141    TTATGAGCATGGTCTCTTCCTGGTTGTGGAAAGAGCTAGGCAAACACAAACGGCCACGAG
1201    TCTGTACCAAAGAAGAGTTCATCAACAAGGTTCGTAGCAATGCAGCATTAGGGGCAATAT
1261    TTGAAGAGGAAAAAGAGTGGAAGACTGCAGTGGAAGCTGTGAACGATCCAAGGTTCTGGG
1321    CTCTAGTGGACAAGGAAAGAGAGCACCACCTGAGAGGAGAGTGCCAGAGTTGTGTGTACA
1381    ACATGATGGGAAAAAGAGAAAAGAAACAAGGGGAATTTGGAAAGGCCAAGGGCAGCCGCG
1441    CCATCTGGTATATGTGGCTAGGGGCTAGATTTCTAGAGTTCGAAGCCCTTGGATTCTTGA
1501    ACGAGGATCACTGGATGGGGAGAGAGAACTCAGGAGGTGGTGTTGAAGGGCTGGGATTAC
1561    AAAGACTCGGATATGTCCTAGAAGAGATGAGTCGCATACCAGGAGGAAGGATGTATGCAG
1621    ATGACACTGCTGGCTGGGACACCCGCATCAGCAGGTTTGATCTGGAGAATGAAGCTCTAA
1681    TCACCAACCAAATGGAGAAAGGGCACAGGGCCTTGGCATTGGCCATAATCAAGTACACAT
1741    ACCAAAACAAAGTGGTAAAGGTCCTTAGACCAGCTGAAAAAGGGAAAACAGTTATGGACA
1801    TTATTTCGAGACAAGACCAAAGGGGGAGCGGACAAGTTGTCACTTACGCTCTTAACACAT
1861    TTACCAACCTAGTGGTGCAACTCATTCGGAATATGGAGGCTGAGGAAGTCCTAGAGATGC
1921    AAGACTTGTGGCTGCTGCGGAGGTCAGAGAAAGTGACCAACTGGTTGCAGAGCAACGGAT
1981    GGGATAGGCTCAAACGAATGGCAGTCAGTGGAGATGATTGCGTTGTGAAGCCAATTGATG
2041    ATAGGTTTGCACATGCCCTCAGGTTCTTGAATGATATGGGAAAAGTTAGGAAGGACACAC
2101    AAGAGTGGAAACCCTCAACTGGATGGGACAACTGGGAAGAAGTTCCGTTTTGCTCCCACC
2161    ACTTCAACAAGCTCCATCTCAAGGACGGGAGGTCCATTGTGGTTCCCTGCCGCCACCAAG
2221    ATGAACTGATTGGCCGGGCCCGCGTCTCTCCAGGGGCGGGATGGAGCATCCGGGAGACTG
2281    CTTGCCTAGCAAAATCATATGCGCAAATGTGGCAGCTCCTTTATTTCCACAGAAGGGACC
2341    TCCGACTGATGGCCAATGCCATTTGTTCATCTGTGCCAGTTGACTGGGTTCCAACTGGGA
2401    GAACTACCTGGTCAATCCATGGAAAGGGAGAATGGATGACCACTGAAGACATGCTTGTGG
2461    TGTGGAACAGAGTGTGGATTGAGGAGAACGACCACATGGAAGACAAGACCCCAGTTACGA
2521    AATGGACAGACATTCCCTATTTGGGAAAAAGGGAAGACTTGTGGTGTGGATCTCTCATAG
2581    GGCACAGACCGCGCACCACCTGGGCTGAGAACATTAAAAACACAGTCAACATGGTGCGCA
2641    GGATCATAGGTGATGAAGAAAAGTACATGGACTACCTATCCACCCAAGTTCGCTACTTGG
2701    GTGAAGAAGGGTCTACACCTGGAGTGCTGTTGTAAAAGTGGATAACGGATCCG
```

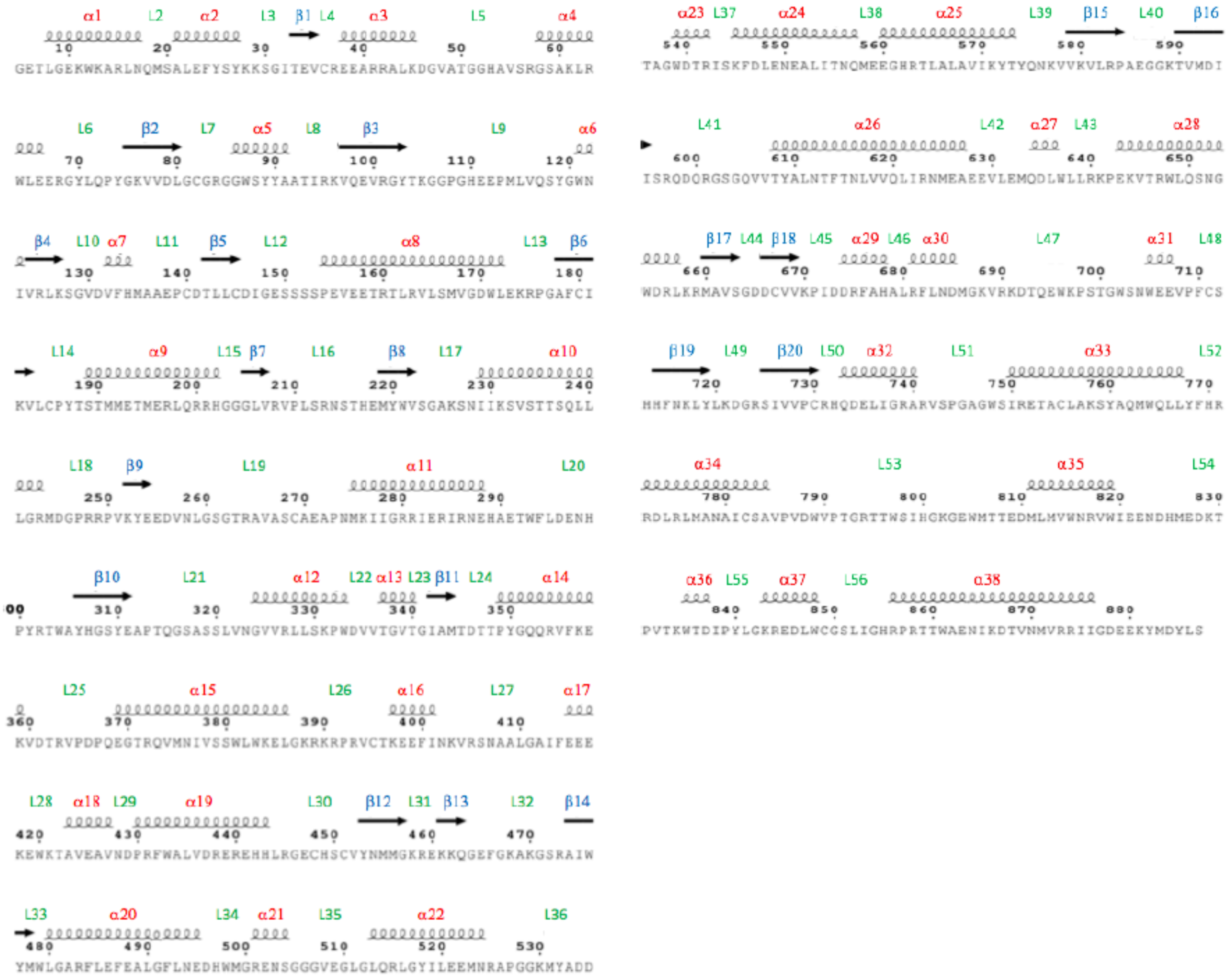

Supplementary Figure 1. Sequence and the secondary structures of the ZIKV NS5 observed in the crystal structure of the ZIKV NS5 protein. The N-terminal 4 residues and C-terminal 16 residues were not resolved in the crystal structure and not shown in the sequence.

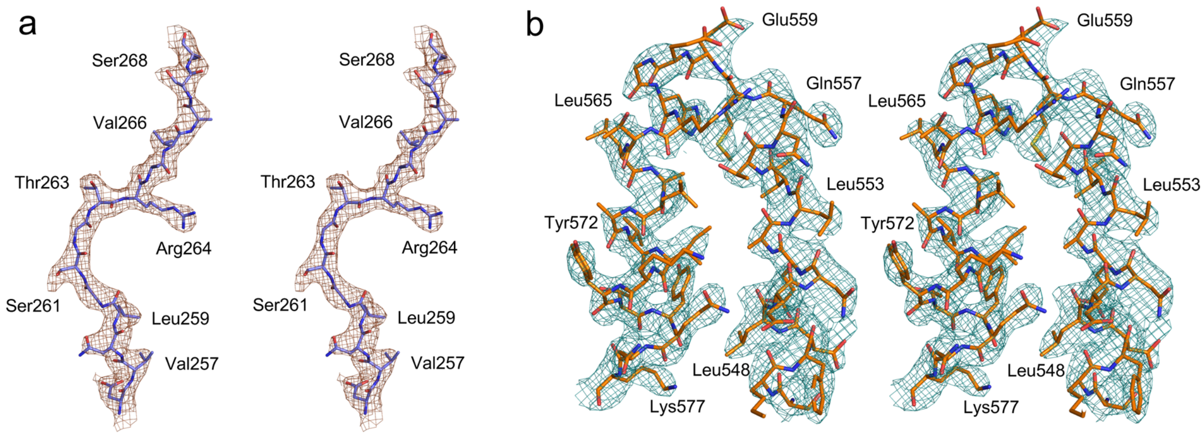

Supplementary Figure 2. Stereo view of representative electron density maps of full-length ZIKV RdRp and the catalytic domain of the protein. (**a**) The *σA*-weighted $2F_o$-F_c map of the linker region between the methyltransferase and catalytic domains of full-length ZIKV RdRp contoured at 1.0σ. (**b**) The *σA*-weighted $2F_o$-F_c map of the catalytic domain of ZIKV RdRp contoured at 1.0σ.

a

```
                        MT residues that
                      interact With RdRp          Linker                   RdRp residues that interact w/ MT
JEV         112 EPMLMQSYGWNLVSLKS 128  266 AVGKGEVHSN 275  350 PFGQQRVFKEK 360 465 GEFGKAKGSR 474 586 AAEGK 590
DENV        112 EPVPMSTYGWNIVKLMS 128  263 HVNAEPETPN 273  347 PFGQQRVFKEK 357 462 GEFGKAKGSR 471 583 TPTG  586
ZIKV MR766  112 EPMLVQSYGWNIVRLKS 128  265 AVASCAEAPN 274  349 PYGQQRVFKEK 359 464 GEFGKAKGSR 473 585 AEGGK 589
```

b

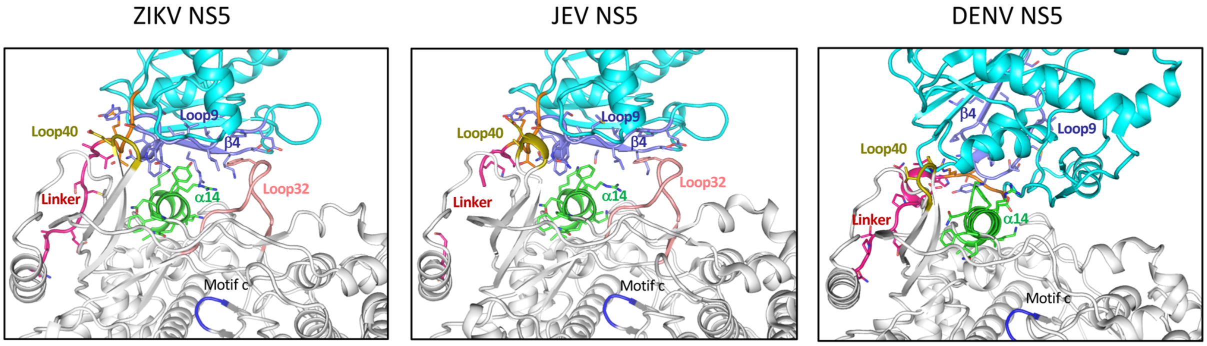

Supplementary Figure 3. The interactions between the MT and RdRp domains in ZIKV, JEV and DENV. (**a**) Sequence alignment of the residues involved in the interactions in ZIKV, JEV and DENV. **(b)** Close-up view of the interactions between the MT (cyan) and RdRp (gray) domains. The sidechains of the residues involved in the interactions are shown as sticks. The MT residues that interact with the RdRp domain are in slate and the linker is in red. RdRp residues that interact with the MT domain are colored green, pink and olive, respectively.

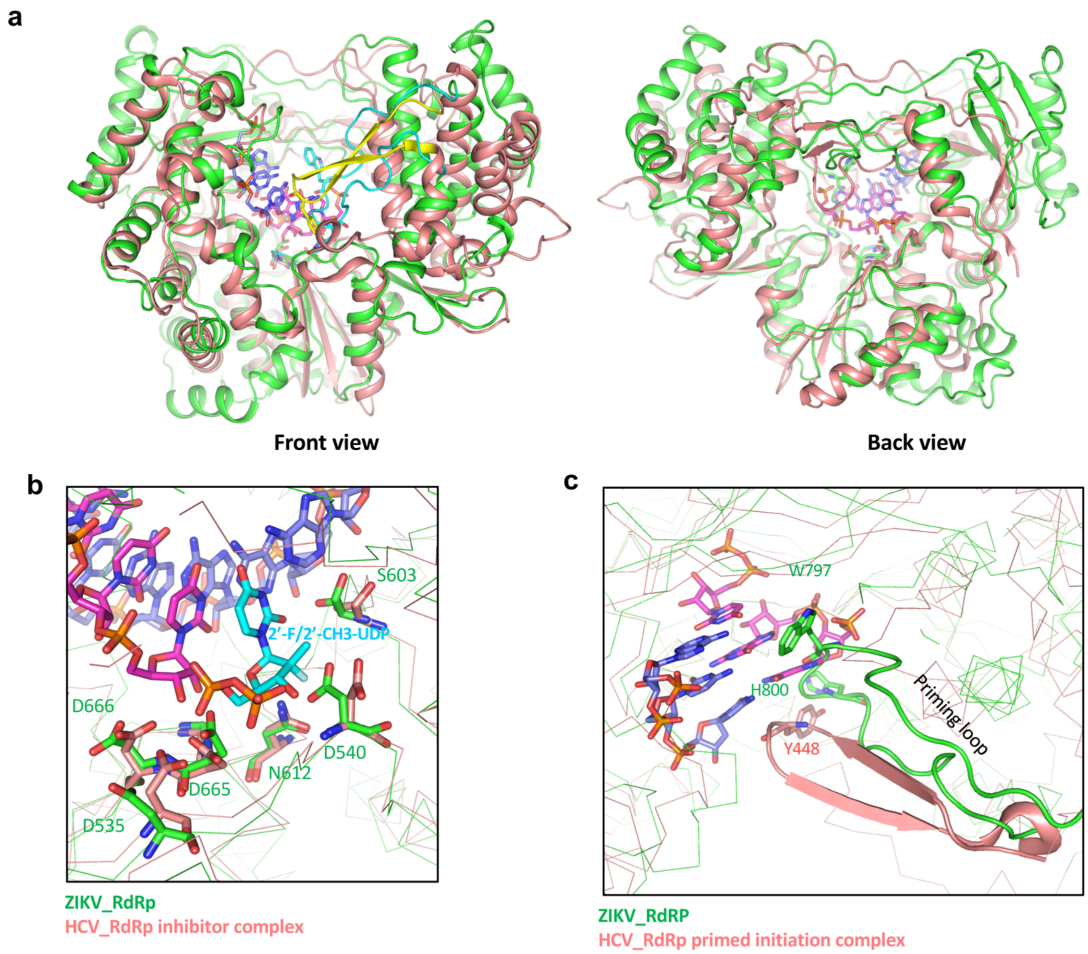

Supplementary Figure 4. Comparisons of the structures of the ZIKV NS5 RdRp with the HCV RdRp/RNA complex. (**a**) Superposition of the structures of ZIKV RdRp and HCV RdRp primed initiation complex (PDB, 4WTL). ZIKV RdRp and HCV RdRp are shown as ribbons colored green and salmon, respectively. The template RNA and the initiation NTPs are shown as sticks and colored slate and magenta, respectively. (**b**) and (**c**) The locations of the priming loops in ZIKV RdRp and in HCV RdRp primed initiation complex (PDB, 4WTL) and HCV RdRp elongation complex (PDB, 4WTA). The priming loops are shown as ribbons and in salmon for HCV NS5 and green for ZIKV NS5.

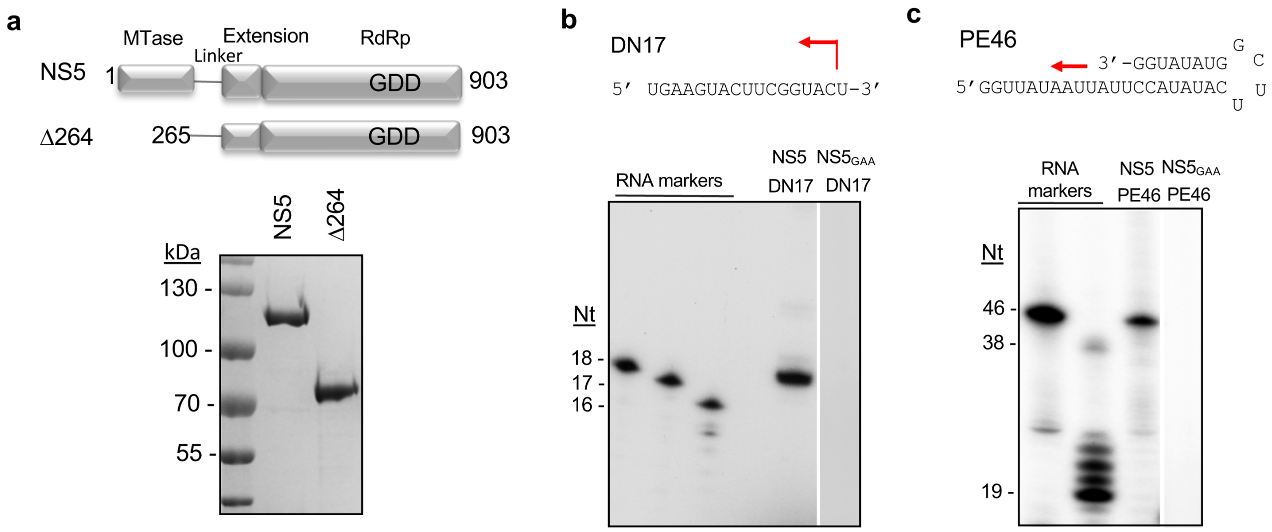

Supplementary Figure 5. ZIKV NS5 possesses RNA polymerase activity in vitro. (**a**) Schematics of full-length NS5 and Δ264 that lacks the MT and SDS-PAGE of purified full-length NS5 and Δ264. The proteins were stained with Coomassie brilliant blue. (**b**) *De novo* initiated RNA synthesis by NS5. The RNA template DN17 that can direct de novo-initiated RNA synthesis. *In vitro* RNA synthesis were performed in reactions containing of 100 ng of either WT NS5 or the catalytically-inactive $NS5_{GAA}$ as described in the Material and Method. The RNA markers were chemically synthesized RNAs of 16, 17, and 18-nt and radiolabeled at their 5' terminus using the T4 polynucleotide kinase and γ-^{32}P-ATP. **C**) Elongative RNA synthesis by NS5. The template used, PE46, forms a self-primed RNA whose 3'-terminal nucleotide can be elongated to form a 46-nt product. The RNA markers of 46- and 19-nt were made using recombinant Con 1 HCV NS5B using the LE19 or PE46 RNA as templates. The RNA bands longer than 19-nt in length were generated by the terminal nucleotide activity of the HCV NS5 protein.

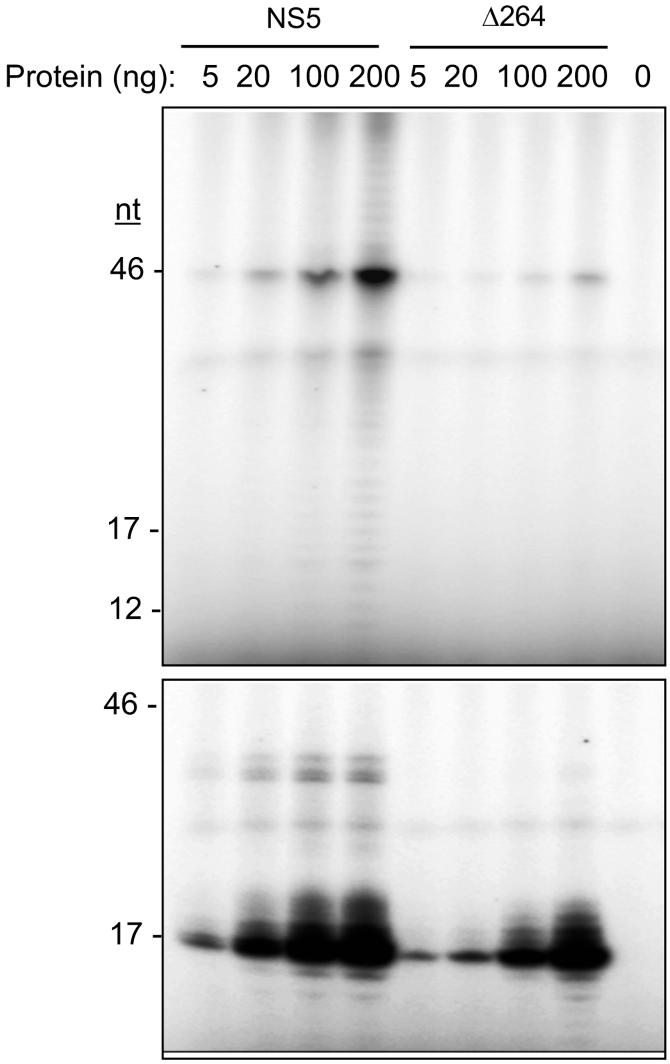

Supplementary Figure 6. Uncropped images of the primer extension and *de novo*-initiated RNA products made by full-length ZIKV NS5 and Δ264. The Δ264 protein that lacks the methyltransferase domain. The primer extension product was made from PE46 and is of 46-nt. The de novo initiated product used the template DN-17, which yields a product of 17-nt. The lengths of the polymerase products were verified as described in Supplemental Figure 5 and corroborated by the positionof fiduciary dyes that were co-electrophoresed with the samples. The difference in the RNA synthesis by NS5 and Δ264 are reproducible in three independent experiments.

a

Sequence	Residues	Row m/z	Error (ppm)
ARLNQMSALEFYSYKKSGITEVCREEAR	14 - 41	659.88	0.31
SGITEVCREEAR	30 - 41	675.33	2.14
GYLQPYGKVVDLGCGR	69 - 84	575.63	1.00
GYLQPYGKVVDLGCGRGGWSYYAATIR	69-95	738.37	0.03
VVDLGCGRGGWSYYAATIR	77 - 95	682.34	-1.96
VVDLGCGRGGWSYYAATIRKVQEVRGYTK	77-105	647.34	0.15
GGPGHEEPMLVQSYGWNIVRLK	106 - 127	823.09	-2.34
MDGPRRPVKYEEDVNLGSGTR	244-264	798.06	2.71
MKIIGRRIERIRNEHAETWFLDENHPYRTW	275-304	1294.99	-1.85
VFKEKVDTRVPDPQEGTR	355 - 372	701.38	-2.10
APGGKMYADDTAGWDTRISKFDLENEALIT	526-555	822.15	0.12
NMEAEEVLEMQDLWLLR	624 - 640	1076.51	-2.97
QSNGWDRLKRMAVSGDDCVVKPIDDRFAH	650- 678	663.93	5.74
LMANAICSAVPVDWVPTGR	777 - 794	1008.51	-1.72
EDLWCGSLIGHRPR	845-858	546.94	-0.29

b

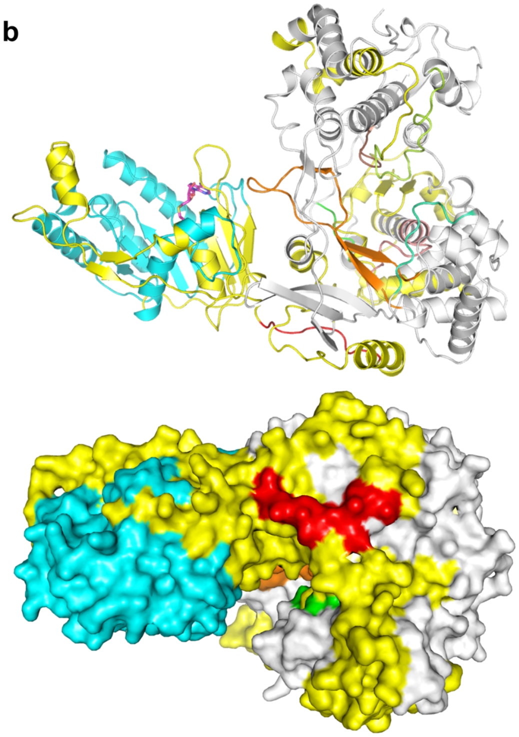

Supplementary Figure 7. The template RNA contacts both the ZIKV MT and the RdRp. (**a**) Peptides from the ZIKV NS5 protein that contact the template RNA. The peptides were identified by a reversible crosslinking and peptide fingerprinting (RCAP) assay.[21] All peptides shown were identified in two independent RCAP assays and also absent in two control reactions where NS5 was not crosslinked to the RNA. The error denotes the difference between the observed and predicted mass of each peptide. (**b**) Locations of the peptides that contact the template RNA mapped onto the structure ZIKV NS5. The MT is colored blue, the RdRp grey, and the peptides that contact the RNA in yellow. Motifs F and D, which were not found to contact the template RNA, are colored brown and red, respectively.

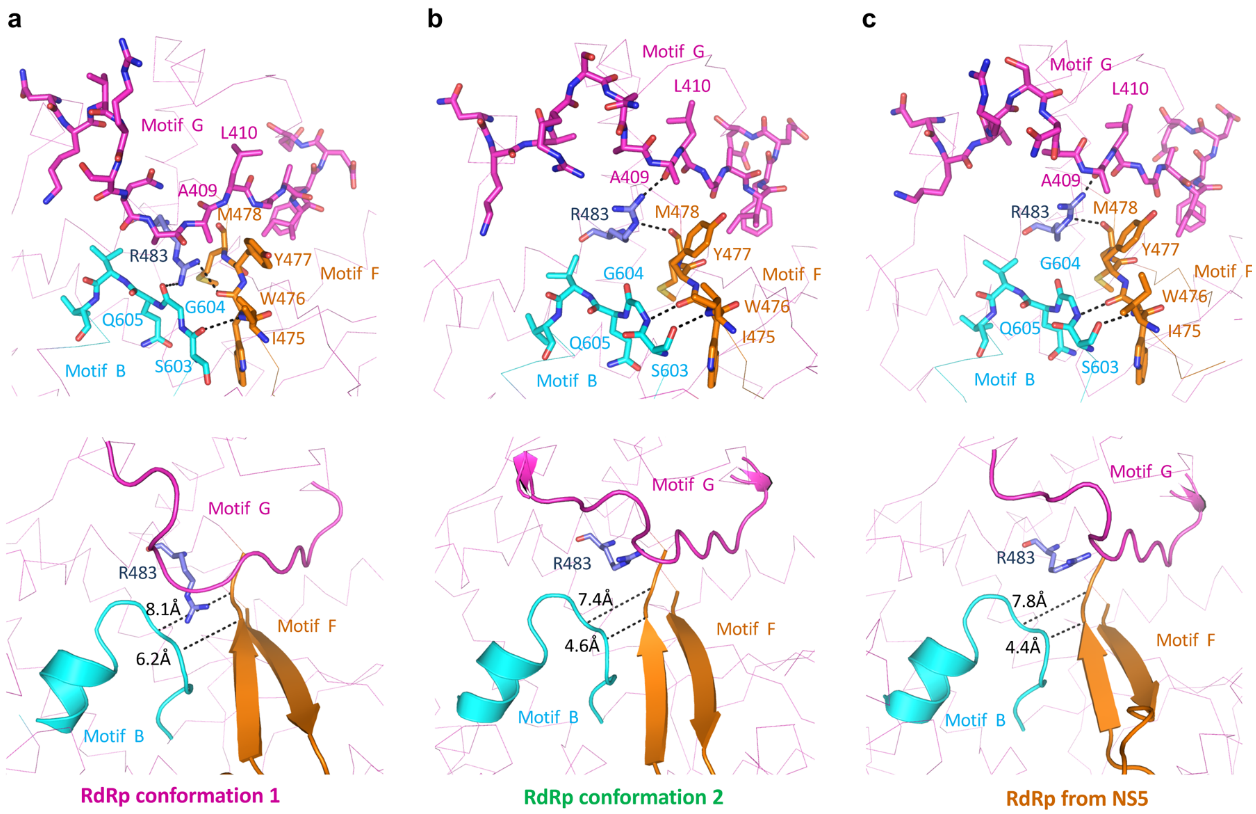

Supplementary Figure 8. Conformation of the ZIKV RdRp in the absence of the MT. (**a**) Motifs in RdRp conformation 1. (**b**) Motifs in RdRp conformation 2. (**c**) Motifs in the full-length NS5. The RdRps are shown as ribbons and in magenta and green. The RdRp from the full-length NS5 is in orange. The shifts of the finger subdomain are labeled. Structures of motifs B, F, and G in isolated ZIKV RdRp (Δ264) and comparison to the structure of full-length NS5. Motifs B, F and G are shown as sticks (up) and ribbons (down) and in cyan, orange and magenta, respectively. Arg483 is shown as sticks and in slate.

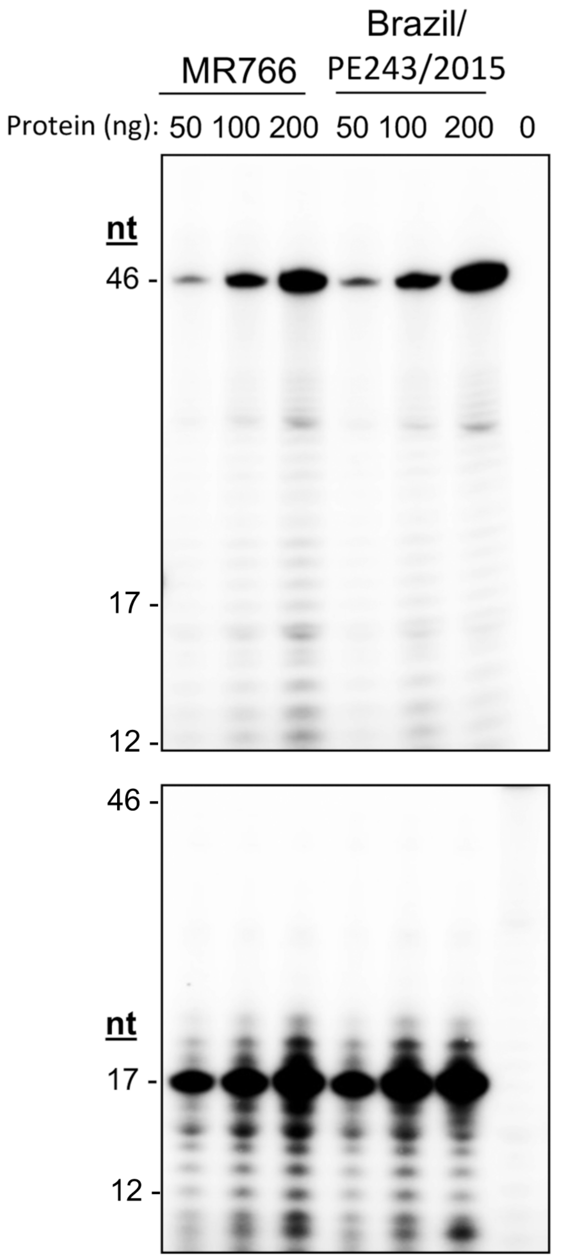

Supplementary Figure 9. Uncropped images of the primer extension and *de novo*-initiated RNA products synthesized by the NS5 proteins MR766 and Brazil/PE242/2015. The primer extension product of 46-nt was made using PE46 as the template. The de novo initiated product of 17-nt used the template DN-17. The lengths of the polymerase products were verified as described in Supplemental Figure 5 and corroborated by the positionof fiduciary dyes that were co-electrophoresed with the samples. The results are reproducible in three independent experiments.
